# Determinants of departure to natal dispersal across an elevational gradient in a long‐lived raptor species

**DOI:** 10.1002/ece3.9603

**Published:** 2023-01-16

**Authors:** Patrick Scherler, Stephanie Witczak, Adrian Aebischer, Valentijn van Bergen, Benedetta Catitti, Martin U. Grüebler

**Affiliations:** ^1^ Swiss Ornithological Institute Sempach Switzerland; ^2^ Department of Evolutionary Biology and Environmental Studies University of Zurich Zurich Switzerland; ^3^ Fribourg Switzerland

**Keywords:** elevational gradient, emigration, food supplementation, *Milvus milvus*, natal dispersal, onset of dispersal, red kite

## Abstract

Attributes of natal habitat often affect early stages of natal dispersal. Thus, environmental gradients at mountain slopes are expected to result in gradients of dispersal behavior and to drive elevational differences in dispersal distances and settlement behavior. However, covariation of environmental factors across elevational gradients complicates the identification of mechanisms underlying the elevational patterns in dispersal behavior. Assuming a decreasing food availability with elevation, we conducted a food supplementation experiment of red kite (*Milvus milvus*) broods across an elevational gradient toward the upper range margin and we GPS‐tagged nestlings to assess their start of dispersal. While considering timing of breeding and breeding density across elevation, this allowed disentangling effects of elevational food gradients from co‐varying environmental gradients on the age at departure from the natal home range. We found an effect of food supplementation on age at departure, but no elevational gradient in the effect of food supplementation. Similarly, we found an effect of breeding density on departure age without an underlying elevational gradient. Supplementary‐fed juveniles and females in high breeding densities departed at younger age than control juveniles and males in low breeding densities. We only found an elevational gradient in the timing of breeding. Late hatched juveniles, and thus individuals at high elevation, departed at earlier age compared to early hatched juveniles. We conclude that favorable natal food conditions, allow for a young departure age of juvenile red kites. We show that the elevational delay in breeding is compensated by premature departure resulting in an elevational gradient in departure age. Thus, elevational differences in dispersal behaviour likely arise due to climatic factors affecting timing of breeding. However, the results also suggest that spatial differences in food availability and breeding density affect dispersal behavior and that their large‐scale gradients within the distributional range might result in differential natal dispersal patterns.

## Graphical abstract

By conducting a food supplementation experiment, we aimed at disentangling food availability and co‐varying elevational gradients affecting the onset of dispersal in juvenile red kites (*Milvus milvus*) along an elevational gradient toward the upper range margin. We found an effect of food supplementation and of breeding density on age at departure, as well as an elevational gradient in timing of breeding. We show that the elevational delay in breeding is compensated by premature departure resulting in an elevational gradient in departure age and therefore conclude that elevational differences in dispersal behavior likely arise due to climatic factors affecting timing of breeding.

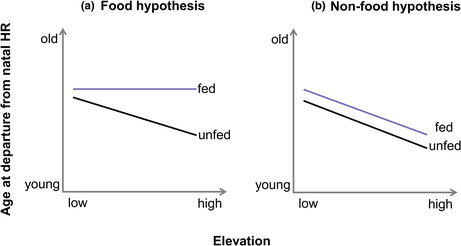

## 1 INTRODUCTION

The onset of natal dispersal represents a pivotal life‐history decision that is shown to affect subsequent dispersal stages (Clobert, 2012; Clobert et al., 2009; Covas & Griesser, 2007) and hence has the potential to shape individual dispersal and life histories (Covas & Griesser, 2007). After gaining independence from their parents, most young animals start natal dispersal and leave the natal home range in order to find their first place of reproduction (Clobert, 2012; Fattebert et al., 2019). Based on life‐history theory, the decision when to depart from the natal home range is subject to a major trade‐off: while delayed dispersal allows individuals to benefit longer from the natal environment and from parental care (Covas & Griesser, 2007; Ekman, 2006), “early dispersers” can start prospecting for future breeding sites early in order to secure vacant resources (Emlen, 1994; Hatchwell & Komdeur, 2000). Departure should therefore occur at the age when the benefits of departure to dispersal exceed the benefits of prolonging the time in the natal home range.

Both, the benefits of delayed dispersal and the benefits of early prospecting depend on a variety of extrinsic factors (Benard & McCauley, 2008; Bowler & Benton, 2005; Clobert, 2012). There is evidence that food availability (Fattebert et al., 2019), conspecific density (Matthysen, 2005), and climatic conditions affect the decision to depart to dispersal (Delgado et al., 2010) and that these effects may differ between sexes (Awade et al., 2017; Eikenaar et al., 2007; Mishra et al., 2018). The spatial heterogeneity of such environmental factors can have important implications for dispersal behavior and thus affects our understanding of populations dynamics, in particular in the face of the current environmental change (Scridel et al., 2018). Environmental factors and the associated habitat quality might be patchily distributed, but often they occur in spatial gradients, and these gradients might co‐vary, resulting in multiple environmental gradients determining spatial variation in dispersal and demography (Burgess et al. 2011). It is important to understand the effect of covarying environmental gradients on dispersal at distributional range edges, because changes in these gradients can considerably affect species distribution (Gaston, 2009). However, we lack a general understanding of how covarying environmental gradients shape gradients in departure to dispersal, that may translate into gradients in settlement success and distances (Clobert, 2012).

Mountain ranges are typically a source of multiple covarying environmental gradients across elevations, often featuring species distributional boundaries (Gaston, 2006). Not only climatic conditions but also resource availability and population structuring can change across elevations. It remains unknown which elevation‐associated gradients affect life‐history traits such as the onset of dispersal (Gaston, 2009; Scridel et al., 2018): For example, climatic gradients might affect departure decisions through elevational gradients of hatching date or food requirements (Badyaev, 1997; Camfield et al., 2010; Scridel et al., 2018), while elevational gradients in habitat composition can affect food availability (Duclos et al., 2019; Gaston, 2006; Sam et al., 2017). Therefore, without experimental approaches, the identification of the separate effects of different elevational gradients on, and their relative contributions to departure decisions remain difficult.

**FIGURE 1:**
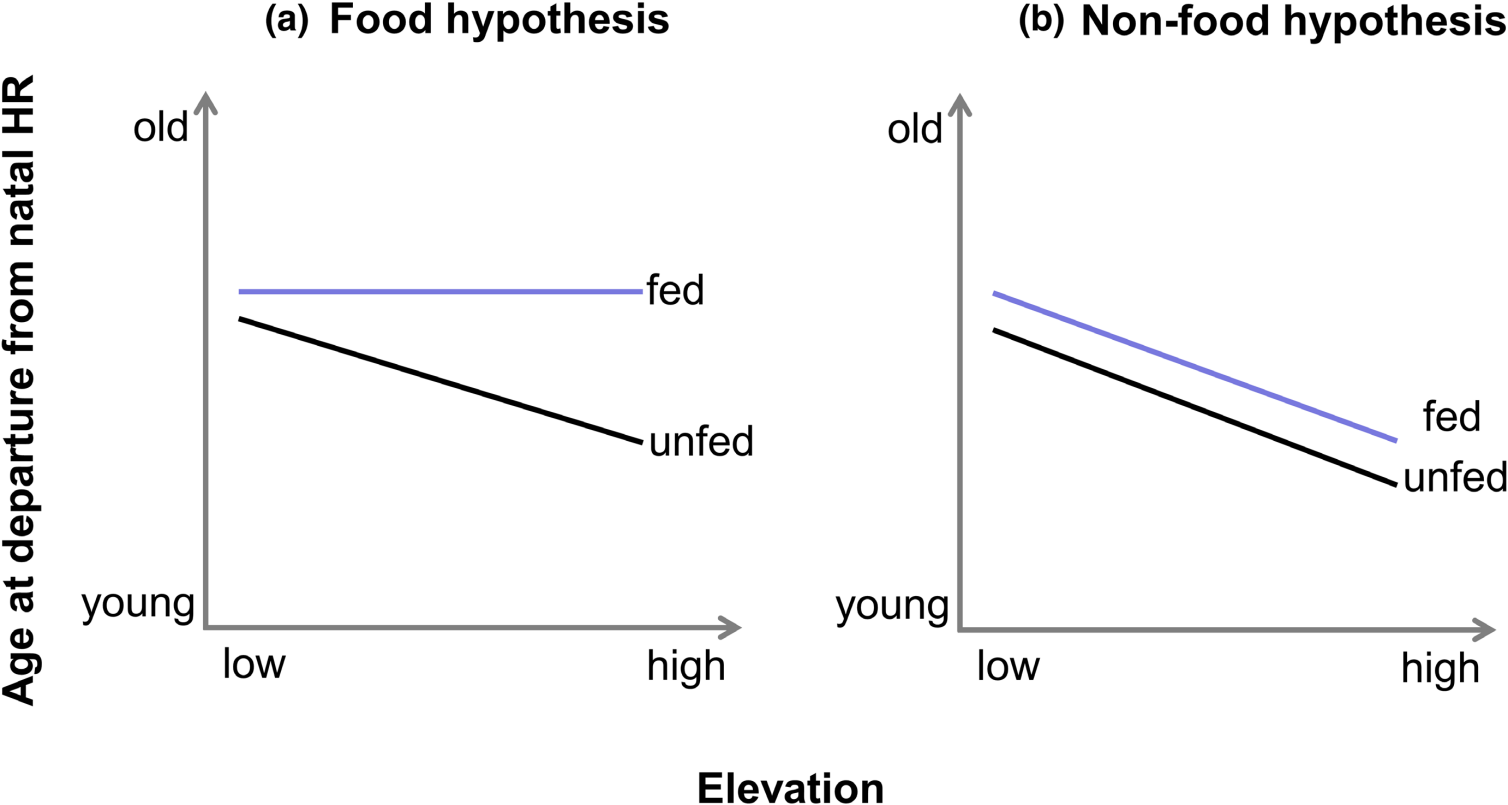
Possible outcomes of the experimental food supplementation of broods across the elevational gradient. If an elevational gradient in departure age is caused by an elevational gradient in food availability during development, we expect the elevational gradient to disappear in *ad libitum* food supplemented individuals because they all developed under similar conditions (“food hypothesis,” a). In contrast, if an elevational gradient in departure age is caused by other environmental factors than food availability during development, we expect an elevational gradient in both fed and unfed individuals (“non‐food hypothesis,” b).

Experimental food supplementation represents a valid approach to investigate condition‐dependent dispersal behavior (Fattebert et al., 2019; Kapota et al., 2016); the additional food is well known to increase body condition (e.g., Bańbura et al., 2011; Catitti et al., 2022; Ruffino et al., 2014). However, it also allows for disentangling the effect of spatial food gradients at natal sites from other environmental gradients on dispersal behavior. If an elevational gradient in the departure decision is caused by an elevational gradient in food availability, we expect the elevational gradient to disappear in *ad libitum* food‐supplemented individuals (“food hypothesis,” Figure 1a). In contrast, if an elevational gradient in the departure decision is caused by other environmental factors, we expect an elevational gradient in departure decisions of both, fed and unfed individuals (“non‐food hypothesis,” Figure 1b). Elevational gradients in timing of breeding and population structuring may covary with food availability and can independently affect departure decisions (Matthysen, 2005; Penteriani et al., 2002). When additionally controlling for these covarying gradients, a food supplementation experiment will provide important insights into the mechanisms of how elevational gradients in particular and environmental gradients in general can affect individual dispersal behavior.

In this study, we investigate the mechanisms underlying the elevational patterns in the decision to depart to dispersal in juvenile red kites (*Milvus milvus*), a medium‐sized, long‐lived raptor species, at the northern slope of the Alps. We tracked large samples of juvenile red kites from fledging onwards, enabling identification of the age at departure across an elevational gradient. We disentangled the effect of potential elevational gradients in food availability from the effect of other elevational gradients on the age at departure by using a food supplementation experiment across elevations. We controlled for effects of gradients in hatching date and breeding density as well as for the potential confounding effects of hatching rank, sex, and potential correlations with body mass at fledging using statistical models. We assume that availability and accessibility of food decreases towards to high margin of the elevational gradient due to associated changes in habitat structure and seasonal weather conditions (Andereggen, 2020). As young individuals may escape the poor conditions at high elevations, we expect that decreasing food resources toward higher elevations result in younger age at departure from the natal home range toward the elevational range margin. Thus, food supplementation should have a larger effect at high than at low elevations (Figure 1). The results of this study will contribute to our understanding of how environmental gradients shape dispersal decisions thereby affecting spatial variation in spatial dynamics and demography.

## 2 METHODS

### 2.1 Study area and species

The red kite is a medium‐sized diurnal raptor species with a pan‐European distribution and an opportunistic feeding behavior (Carter, 2007). In Switzerland, the species showed a rapid recovery from under 100 breeding pairs in the 1950 s to between 2800 and 3500 breeding pairs in 2018 (Aebischer & Scherler, 2021; Knaus et al., 2018). The study was conducted in 2016 and 2017 in western Switzerland in a study area of 387 km^2^ within the Cantons of Bern and Fribourg. It comprises an elevational gradient from 482 to 1763 m.a.s.l., reaching from the Swiss Plateau to the subalpine range. The study area is characterized by agriculture (56.25%) and managed forests (26.95%). The agricultural parts are dominated by crops and dairy farming, as well as meat production, resulting in large areas with meadows (StatA, 2017) and, thus, possible red kite breeding territories. In our study population, red kites breed in small forest patches in heterogeneous agricultural landscapes where they find natural prey, but also profit from anthropogenic food provision (Cereghetti et al., 2019; Heiniger, 2020). While agricultural areas at low elevations harbor crop farming, agricultural areas at high elevations are almost exclusively used for dairy farming and meat production. This gradient in agricultural land use also affects the prey spectrum of red kites, which shows an increasing proportion of rodents at higher elevations (Andereggen, 2020). The study area harbors approximately 130 red kite breeding pairs per year, representing a high breeding density compared to other breeding areas in Europe (Aebischer & Scherler, 2021). The lowest brood was recorded at 524 m.a.s.l, the highest brood at 1129 m.a.s.l. (mean = 765 m.a.s.l ± 123 SD, *n* = 171).

### 2.2 Tagging and individual measurements

Red kite territories were identified by a standardized territory survey in 2.5 x 2.5 km^2^ squares. We repeatedly visited territories until a nest was located, and we then monitored nests by observations to confirm incubation and hatching of the nestlings. If the growth of brown covert feathers was observed (nestling age 15–20 days), we climbed the nest to ring and measure nestlings. Based on wing length measurements, we approximated age using a generic growth curve by Aebischer (2009) in order to assess ideal tagging date at the age of 38–42 days. The elevation of the nest was calculated based on swissALTI^3D^ DEM (© swisstopo 2017). During the 2 years, a total of 239 nestlings were equipped with solar powered GPS‐GSM‐UHF transmitters (Ecotone SKUA/CREX type) using backpack style diagonal‐loop harnesses (Kenward, 1985, 2001) with 6.4 mm wide tubular Teflon tape (Bally Ribbon Mills), reinforced with an internal 1.5 mm Polypropylene string. The weight of the transmitters including the harness was 26 g, which corresponds to 2.2%–4.0% of the body weight at tagging. Nestlings were GPS‐tagged at the age of 39.0 ± 3.4 SD days (range = 27–48 days, *n* = 239) when they reached at least 670 g in body weight in order to fit the harness properly, but were not yet at risk of escaping capture due to impending fledging (Bustamante, 1993). GPS transmitters provided hourly locations with two different duty cycles from February to September (03:00 to 21:00 UTC) and from October to January (5:00 to 19:00 UTC), in order to fully cover daylight periods in central and southern Europe, while optimizing battery recharging. All data were then transmitted via GSM and automatically stored on the Movebank data repository (movebank.org).

At tagging, we measured body mass, tarsus length, and the length of the eighth primary feather. This allowed us to determine the exact hatching date based on a study area‐specific primary feather growth curve (Nägeli, 2019). In addition, we determined hatching rank from ordering wing length measures among siblings. We estimated a body mass‐based body condition measure using residuals from a mass–size relationship (for details, see supporting information S3). Additionally, we recorded the number of siblings and collected a small blood sample from the brachial vein. We determined sex based on blood samples preserved on filter papers. DNA was extracted and purified using QIAGEN DNeasy and Tissue kit and the sex identified by PCR amplification of the CHD1 gene using primers 2550 and 2718 as described in Fridolfsson and Ellegren (1999).

### 2.3 Supplementary feeding experiment

In order to disentangle elevational gradients of food availability from other environmental gradients across elevations, we conducted a supplementary feeding experiment. We selected nests in a stratified design along the elevational gradient (equal proportion of supplemented and unsupplemented nests for every 100m elevational band). Every other day, we placed five dead day‐old chickens per adult and per nestling <10 days, and 10 chickens per nestling >10 days of age on wooden platforms located 20–200 m away from the target nests until the nestlings were close to fledging, that is, approximately 52 days old. Thus, food availability during nestling development, but not in the post‐fledging period was experimentally increased. The provided food exceeds the known daily food requirements of red kite nestlings (approx. 300g per day, Wasmund, 2013) and, hence, we created a quasi‐*ad libitum* supplementation setting. We assessed whether the food supplementation was accepted by observing the platform and nest shortly after a feeding event using either binoculars or video cameras.

### 2.4 Breeding density

Conspecific territory density, a measure of breeding density, was calculated based on the territory survey adapting the method described in Knaus et al. (2018). Territory density was represented by the number of territory centroids in a radius of 2 km from the nest; data gaps at the edge of the study area were corrected proportionally.

### 2.5 Age at departure

We estimated individual departure from the natal home range based on (a) a radius surrounding the nest, and (b) a duration in which the individual continuously had to stay outside the radius. This spatio‐temporal threshold assumes that once the individual stays outside the radius longer than the temporal threshold, it can be considered as having departed from the parental home range. We used a 2 km radius surrounding the nest as spatial threshold. This value was based on known breeding home range sizes of adult male breeding birds in our study area (Baucks, 2018). Daily food intake is likely to be crucial for juvenile red kites; hence, we assumed that individuals spending more than 2 days outside the radius were independent and definitively departed from their parental home range (Wasmund, 2013). We validated this assumption by choosing different temporal thresholds (1/2/3/5 and 9 days) in order to evaluate how this would influence the estimated age at departure from natal home range (see supporting information S1). Qualitative analyses of entire family trajectories (*n* = 5) showed that departures from the natal home range exceeding 2 days are abrupt large‐scale movements and returns to the 2‐km radius for an entire day occur rarely (see supporting information S2). We estimated the post‐fledging dependence period by subtracting fledging age from the age at departure, assuming a mean fledging age of 55 days (Bustamante, 1993).

Non‐breeding red kites tend to form social roosts to spend the night. If social roosts occur near the natal home range, independent individuals show higher probability to incidentally return to the natal home range during roosting. The distance to the closest communal roost was therefore considered as a control variable in order to account for confounding effects of roosts located close to natal nest. The roosts were identified based on night‐time positions of tagged non‐breeders (including juveniles) in the study area with an external buffer of 10 km. We calculated the number of revisits in a radius of 50 m around each position for each year from July to September using the R‐package “recurse” (Bracis et al., 2018). We selected the top 5% of the positions with highest re‐visitation rates and k‐mean clustered them into 10 clusters per year representing the 10 most highly visited roosting locations. We then calculated the distance from the natal nest to the closest roosting location for the respective year.

### 2.6 Statistical analyses

If juveniles died or if their transmitters stopped working before departure from the natal home range, they were excluded from the analysis (*n* = 64 out of a total of 239). In addition, individuals that did not depart from the natal home range before starting migration were excluded (*n* = 17) as start of migration is likely mediated by a different set of factors than the departure to dispersal. This resulted in a total of 158 juveniles which were included in further analyses.

All spatial and statistical analyses were performed using the statistical software R (R Core Team, 2019). We divided the modelling process into three sequential steps: 1) We modelled effects of elevational gradients on age at departure. As we expected that elevation might show indirect effects on age at departure through elevational gradients, we 2) modelled effects of elevation on parental brood timing and population structure (territory density and distance to roost site). To address potential confounding condition‐dependent effects on age at departure, we 3) assessed the effect of body condition on age at departure.

In order to quantify the effect of elevational gradients on age at departure, we included the effects of elevation, food supplementation, and their interactions as fixed effects in a linear mixed effect model (R‐package “lme4,” Bates et al., 2015), while considering the effect of territory density and controlling for sex, hatching rank, distance to roost site, and year. No correlations *r* > .7 were found among fixed effects used in the model and, hence, all variables remained in the model (Tabachnick & Fidell, 2019). In order to account for the dependent structure of siblings originating from the same brood, we included brood ID as a random effect with restricted maximum‐likelihood (REML) estimation of the associated variance component. All continuous variables were scaled and centered. We included all possible two‐way interactions and removed non‐significant interactions in a stepwise procedure. Non‐significant fixed effects were retained in the model. We calculated 95% credible intervals (CrI) for the estimates using a simulated posterior distribution of 2000 simulations (R‐package “arm,” Gelman & Hill, 2009). Finally, we tested the significance of the random effect comparing the final model to a linear model without random error structure using a likelihood‐ratio test (R‐package “RLRsim,” Scheipl et al., 2008).

To quantify indirect effects on age at departure through elevational gradients in parental brood timing and population structure, we tested for effects of elevation on hatching date, territory density, and distance to roosting site. We quantified effects on hatching date using a linear mixed effect model including elevation, year and territory density as additional fixed effects and brood ID as random effect. The effect of elevation on territory density and distance to roosting site was modeled using linear models. Apart from elevation we additionally included year as a fixed effect in the territory density model and territory density, as well as year as additional fixed effects in the distance to roosting site model. We did not consider any interactions in those models. We performed a path analysis to assess direct and indirect effects of elevation and hatching date on age at departure (Grace, 2006).

To investigate the effects of food supplementation on body mass at fledging, the likely mechanism of condition‐dependent departure due to experimental treatment, we first assessed effects of the feeding treatment and elevation on body condition. In a second step we analyzed the effect of body mass on age at departure (for details, see supporting information S3).

## 3 RESULTS

In total, values of departure age from 158 fledglings of 105 broods were available, including 50 individuals of 33 broods with food supplementation (22 females, 28 males) and 108 individuals from 72 broods without food supplementation (51 females, 57 males). The median age at departure was 83 days (mean age = 84.04 days ±10.16 SD, *n* = 158). There was considerable variation in the age at departure among individuals ranging from 61 to 115 days (Table S1). Thus, the post‐fledging dependence period ranged from 6 to 60 days. Departures occurred in a span of 65 days between July 7th and September 10th (median = August 1st).

**FIGURE 2:**
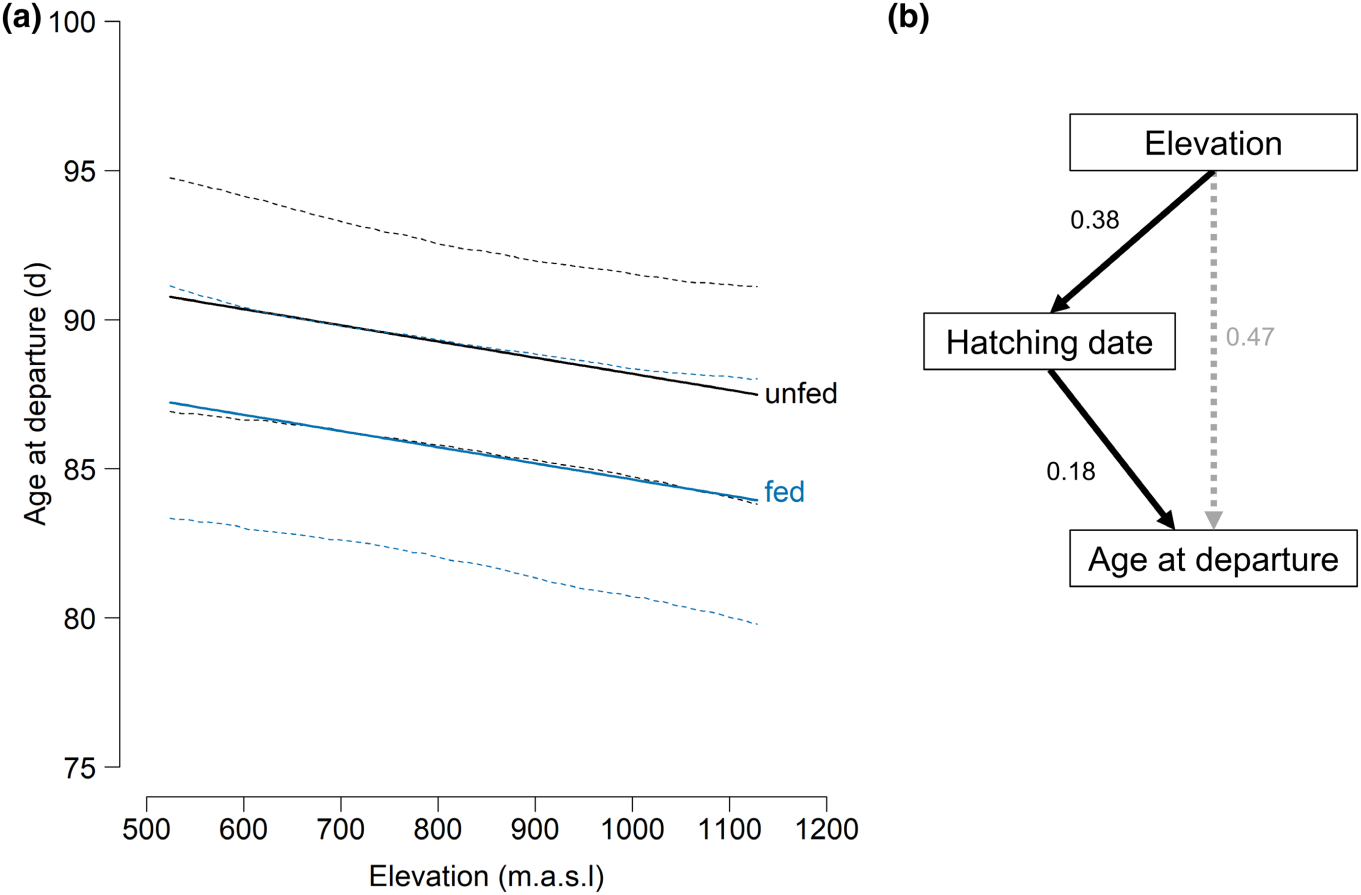
Effects of elevation on age at departure. (a) Predicted values of the indirect effect of elevation over hatching date on age at departure in relation to food supplementation for 50 fed (blue), and 108 unfed juveniles (black) based on estimates of two linear mixed effect models (effect of elevation on hatching date and effect of hatching date on age at departure). Model predictions for male individuals with hatching rank 2nd‐4th born in 2017 were shown. Other predictors were set to their mean. 95% credible intervals (CrI) are shown by dashed lines. (b) Path analysis with effects sizes of direct and indirect effect of elevation on age at departure. The direct effect of elevation (dashed) was not statistically significant.

**FIGURE 3:**
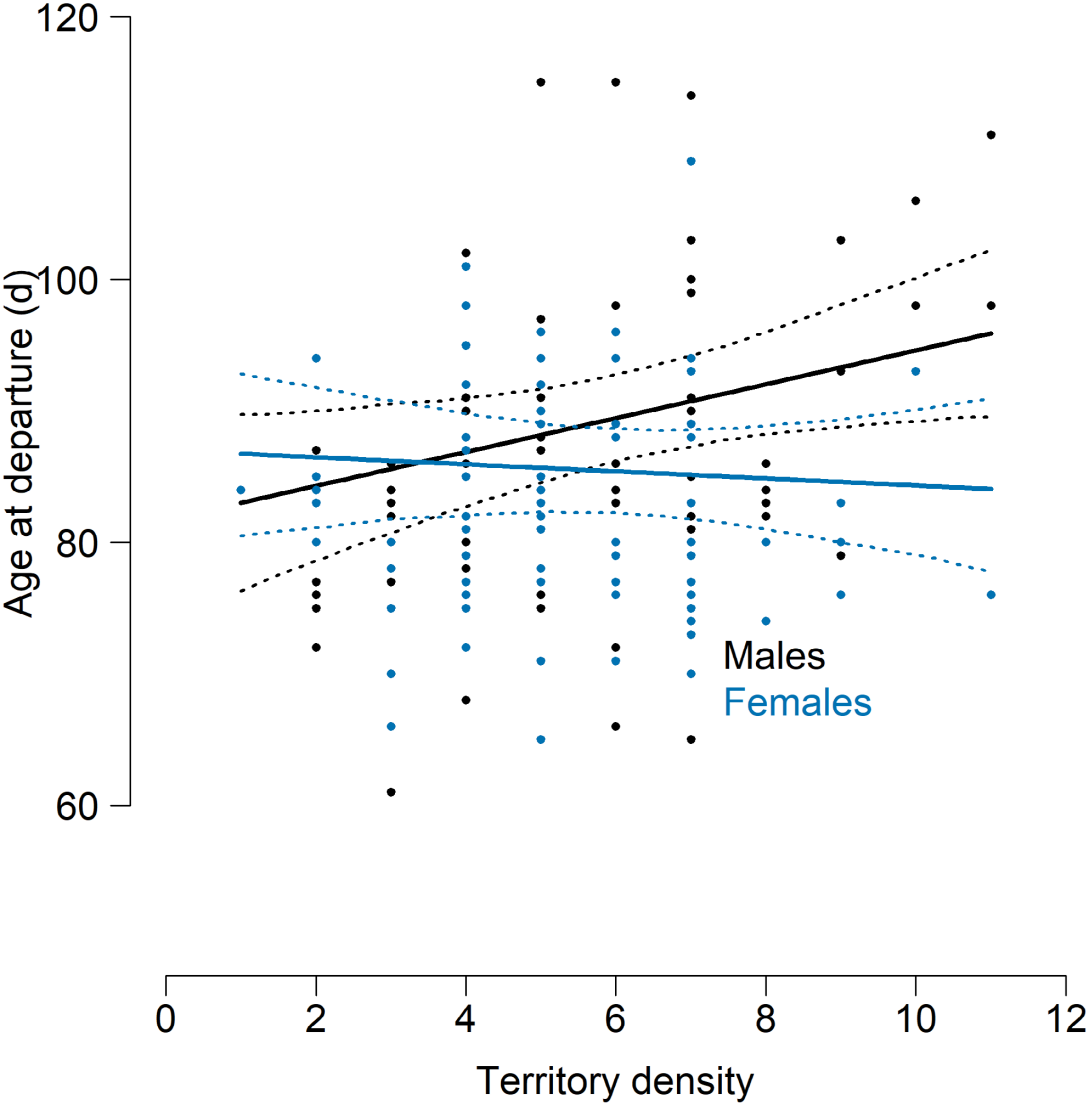
Model predictions (lines) and raw data points (dots) of age at departure in relation to territory density for 73 female (blue) and 85 male juveniles (black) juveniles. Model predictions are shown for unfed individuals with hatching rank 2nd–4th born in 2017. Other predictors were set to their mean. 95 % CrI are indicated by dashed lines.

We found neither a direct effect of elevation nor an interactive effect of any explanatory variable with elevation on age at departure (Table 1). Juveniles from food‐supplemented broods showed an earlier departure age than juveniles from control broods (difference = 3.52 days ± 1.76 SE; Figure 2) at both, high and low elevations. However, since we did not find an interaction between elevation and supplementary feeding, departure age did not change in relation to elevation due to an elevational gradient in food availability, supporting the non‐food hypothesis (Figure 1b). We found an effect of hatching date on age at departure. Late hatching dates resulted in younger age at departure than early hatching dates (Table 1, Figure 2). Sexes differ in age at departure in situations of high territory density, with females showing earlier departure than males (Figure 3). We found neither an effect of size‐corrected fledging body mass on age at departure (Supporting information; Table S3.2), nor an effect of feeding treatment on size‐corrected fledging body mass (Supporting information; Table S3.1).

**TABLE 1:** Model estimates of the linear mixed effect model investigating factors affecting age at departure.

Variable	Estimate	SE	95% CrI
Intercept	**79.47**	**1.83**	**75.74–83.30**
Feeding treatment [fed]	−**3.52**	**1.76**	−**6.85–**−**0.28**
Hatching date	−**2.14**	**0.97**	−**4.00–**−**0.18**
Hatching rank [2nd–4th hatched]	1.49	1.70	−1.80–5.03
Hatching rank [singleton]	0.24	1.96	−3.68–4.25
Elevation	0.39	1.00	−1.52–2.56
Sex [Male]	**3.26**	**1.47**	**0.50–6.17**
Distance to roosting site	−**2.90**	**1.11**	−**5.14–**−**0.78**
Year [2017]	**4.57**	**1.87**	**0.90–8.27**
Territory density	−**4.99**	**1.75**	−**8.57**–−**1.55**
Year [2017] * Territory density	**4.44**	**1.79**	**0.94–8.03**
Sex [Male] * Territory density	**3.38**	**1.48**	**0.48–6.34**

*Note*: Brood ID was included as random effect (*n* = 158 individuals from 105 broods).

Significant effects are highlighted in bold.

Random effect variance [95% credible interval]: Brood 3.20 [2.68, 3.76], LRT 0.13, *p* = .23.

**FIGURE 4:**
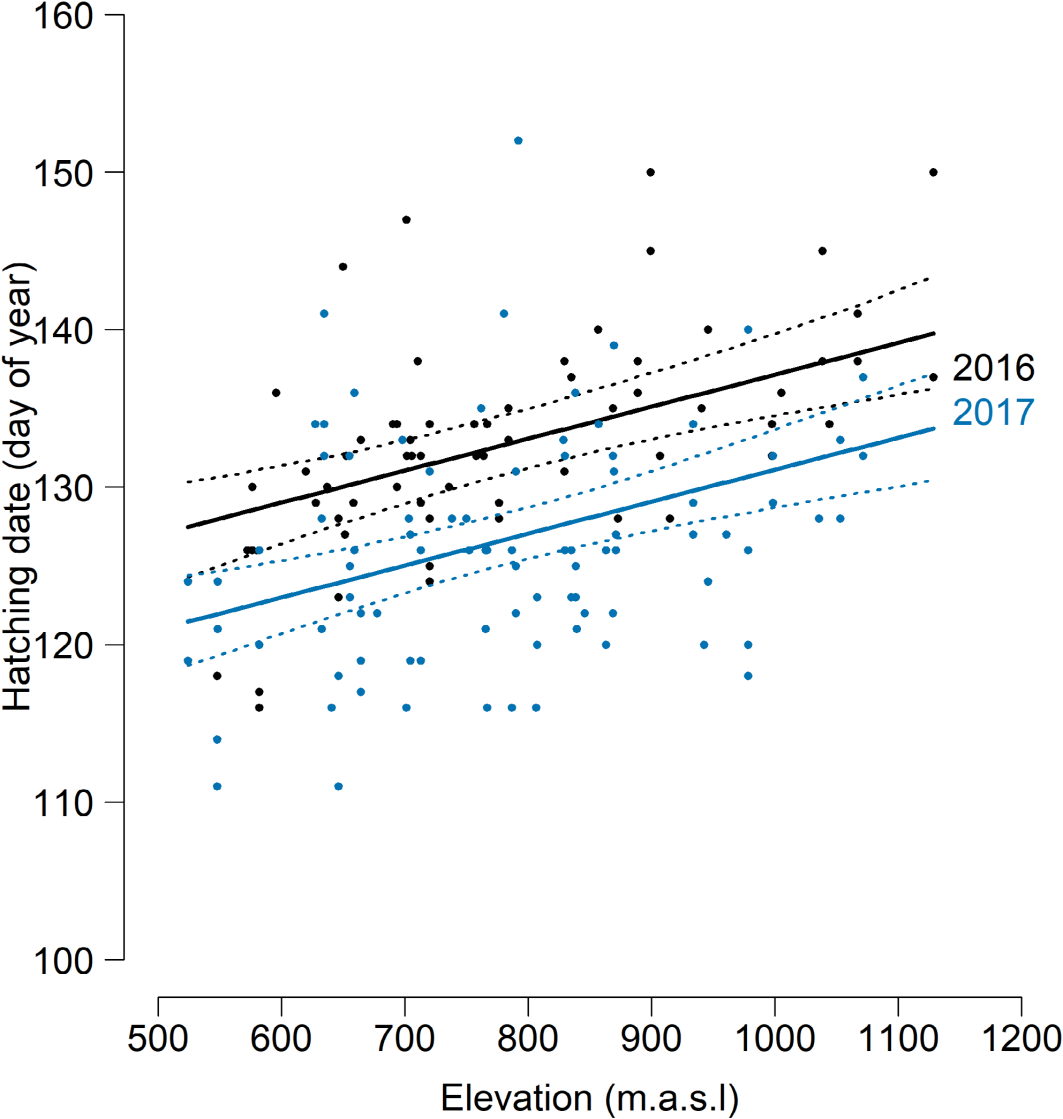
Model predictions (lines) and raw data points (dots) of hatching date in relation to elevation for 67 juveniles born in 2016 (black) and 91 juveniles born in 2017 (blue). Model predictions for individuals at average territory density are shown. 95% CrI are indicated by dashed lines.

The models investigating factors affecting hatching date and territory density indicated an elevational gradient in hatching date, but not in territory density (Table 2). Hatching occurred later at high than low elevations (on average 3.03 days later per 100 m elevation gain; Figure 4). Thus, though elevation showed no direct effect on age at departure, juveniles at high elevations showed a younger departure age than juveniles at low elevations (Figure 2a) due to the elevational gradient in hatching date.

**TABLE 2:** Model estimates of the linear mixed effect model investigating the factors affecting hatching date and the linear models investigating factors affecting territory density and distance to roosting site.

Variable	Hatching date^a^			Territory density^b^			Distance to roosting site^b^		
	Estimate	SE	95% CrI	Estimate	SE	95% CrI	Estimate	SE	95% CrI
Intercept	**132.71**	**0.92**	**130.81–134.6**	**4.80**	**0.26**	**4.31–5.29**	**4039.44**	**259.19**	**3503.43–4559.92**
Elevation	**2.95**	**0.64**	**1.65–4.23**	0.30	0.17	−0.03 – 0.63	**−1499.25**	**167.11**	**−1835.98 – −1169.58**
Territory density	−0.69	0.68	−1.98 – 0.64	—	—	—	**−743.20**	**173.8**	**−1084.19 – −391.04**
Year [2017]	**−5.99**	**1.28**	**−8.54 – −3.56**	**1.23**	**0.34**	**0.57–1.9**	−526.15	347.21	−1210.28 – 138.64

*Note*: Brood ID was included as random effect in the hatching date model (*n* = 158 individuals from 105 broods).

Significant effects are highlighted in bold.

^a^ Random effect variance [95% CrI]: Brood 5.26 [4.73, 5.84], LRT 26.50, *p* > .001.

^b^ linear model without random error structure.

As expected from the methodology to identify departure age, distance to the next roosting site showed an effect on departure age: individuals fledged from a nest in the proximity of a roosting site departed later than individuals fledged from a nest far from roosting sites. We also found an elevational gradient in distance to roosting sites suggesting that at high elevations roost sites were closer to nesting sites than at low elevations (Table 2).

## 4 DISCUSSION

Dispersal behavior might change over the elevational gradient due to changing environmental conditions. In this study, we found that food availability and territory density affected the start of dispersal in terms of departure age. However, these habitat and population characteristics did not change over the elevational gradient. In contrast, hatching date was later at high than at low elevations and late hatched juveniles departed at younger age than early hatched juveniles. This chain of effects resulted in earlier departure at high elevations. In our study area, the gradient in the start of dispersal toward the elevational range margin was mainly due to the elevational change in the timing of breeding. However, the results suggest that in red kite populations, gradients in food availability and population structure can result in gradients in the onset of dispersal and that all these gradients may drive spatial gradients in dispersal behavior.

Food‐supplemented juveniles in our study departed at a younger age than control juveniles. However, the effect of food supplementation did not change along the elevational gradient. These results support an effect of food availability during the nestling period on the age at departure to dispersal as well as the non‐food hypothesis (Figure 1b) for the underlying mechanism of an elevational gradient in dispersal behavior. Thus, food availability in our study area seems not to show an elevational gradient, but a patchy spatio‐temporal variation. A missing elevational gradient in food availability might be due to the opportunistic foraging behavior of red kites, potentially resulting in two adjustments across the elevational gradient. First, the elevational gradient may affect the availability of different prey types, while the total food availability remains stable. This is supported by preliminary results showing that provisioning of different prey types to the nest changed across the elevational gradient (Andereggen, 2020). Second, anthropogenic feeding of red kites is highly prevalent in our study area, in particular at places far from closed settlements (Cereghetti et al., 2019). Thus, while small carcasses originating from human‐induced mortality (i.e., collisions) might be more important at low than at high elevations (Welti et al., 2019), intended feeding of raptors might be more important at high elevations.

We found neither an effect of food supplementation on size‐corrected body mass, a common measure for body condition (Peig & Green, 2010), nor an effect of body mass on departure age. In red kites, food supplementation during the nestling period mainly affected survival of nestlings and growth rate during the exponential part of the growth curve, rather than the final fledging body mass (Nägeli et al., 2022). Nonetheless, we recently showed that the feeding treatment strongly influenced the stress physiology of red kite fledglings (Catitti et al., 2022). Thus, our results suggest that condition‐dependent departure decisions in red kites are due to physiological components of body condition rather than due to the body mass component. A careful use of different body condition measurements and an increased awareness of the different components of body condition affecting condition‐dependent behaviors is therefore needed.

The early departure under food‐rich conditions allows individuals to prolong pre‐migration prospecting duration, which in turn might affect future dispersal and settlement decisions (Azpillaga et al., 2018). This is in accordance with and thus a possible mechanisms resulting in the “silver spoon” hypothesis that predicts that individuals experiencing good natal habitat conditions perform better in their adult life than individuals experiencing poor conditions (Pigeon et al., 2019). If this is the case, red kites in our study population are likely subject to a timing trade‐off between the time spent in the natal home range and time invested in pre‐migration prospecting, as timing of autumn migration is highly fixed (Scherler, 2020). As age at departure includes both, the age at fledging and the post‐fledging dependence period, higher developmental rates in the nest are the likely reason for our experimental effect which is further supported by the effect of food supplementation on body condition during nestling growth (Nägeli et al., 2022). Our experiment quantifies the separate effect of food availability during the nestling period on the start of dispersal after the post‐fledging dependence period. Thus, it remains unclear how food availability during the post‐fledging dependence period might additionally affect the age at departure (but see Bustamante (1994)).

We also found the age at departure to be influenced by hatching date. Departure at a younger age in late‐hatched individuals seems to compensate for the late timing. Hatching date was later at high than at low elevations and affected age at departure indirectly through its elevational gradient. The elevational timing gradient is most likely caused by longer snow cover and lower temperatures at high elevations (Frigerio et al., 2021; Lehikoinen et al., 2011). This result suggests a synchronization of the start of dispersal. Late individuals and individuals from high elevations seem to forfeit the benefits of a prolonged stay in the natal home range in order to avoid lagging behind their conspecifics in the start of dispersal. The elevational change in the trade‐off leading to the departure decision suggests considerable benefits of a prolonged prospecting period (Weston et al., 2018) or considerable costs of starting dispersal later than other juveniles in the population (Bonte et al., 2012; Emlen, 1994). Alternatively, the earlier departure might also be caused by the decision of late breeding parents to shorten the post‐fledging care period (Bustamante, 1993, 1994; Bustamante & Hiraldo, 1993). In both cases, juveniles depart later and therefore potentially pay costs of late departure such as decreased survival and dispersal performance.

Age at departure from natal home range was decreased especially for females in high compared to low breeding densities. While males overall depart at older age, this difference disappears at sites of low breeding density. Breeding density did not change over the elevational gradient. The results suggest that males in high‐density areas might benefit more from staying in the natal home range than females. We showed that males are more likely to exhibit philopatric post‐migration prospecting behavior than females (Scherler, 2020). We therefore suggest that for males in high‐density situations gathering information in and around the natal home range for a longer period might be beneficial for philopatric settlement decisions (Giraldeau et al., 2002; Pärt et al., 2011). Two mutually non‐exclusive mechanisms might be involved. First, high‐density areas likely show increased competition for territories (Matthysen, 2005; Penteriani et al., 2011), and therefore a higher male investment into information about territory owners and their breeding habitat, or a longer protection from competing conspecifics might be beneficial for the future settlement success close to the natal site (Penteriani et al., 2011; Reed et al., 1999). Spatial gradients in breeding density then are expected to be the base for gradients in male competition for settlement, and thus for spatial sorting (Doligez et al., 2004; Matthysen, 2005). Second, due to higher fitness benefits of settling in high‐density areas (Komdeur, 2001), males from high‐density areas are more likely to develop a philopatric dispersal strategy than males from low‐density areas (Shutler & Weatherhead, 1991). High‐density areas then would contribute more to female‐biased dispersal than low‐density areas and gradients in breeding densities would result in gradients of the female‐biased dispersal pattern.

In conclusion, we show that in our study species elevational differences in dispersal behavior likely arise due to climatic factors affecting timing of breeding and that the elevational delay in breeding is compensated by premature departure resulting in an elevational gradient in departure age with potential effects on later dispersal stages and future performance. In this way, parental settlement decisions may carry over to the next generation's dispersal behavior. The food and the timing effect on departure age suggests considerable benefits of an early timing of departure. Both departure age and departure timing seem to be important for fitness‐relevant mechanisms operating during the subsequent prospecting phase of dispersal. While departure age (i.e., premature departure) might affect competitiveness of individuals (Emlen, 1994; Komdeur, 2001), timing of departure might affect the duration of prospecting before migration and the start of prospecting relative to other juvenile individuals in the population. The results also suggest that spatial differences in food availability and breeding density affect dispersal behavior and that their large‐scale gradients within the distributional range might result in differential natal dispersal patterns.

## AUTHOR CONTRIBUTIONS

**Patrick Scherler:** Conceptualization (equal); data curation (equal); formal analysis (lead); funding acquisition (supporting); investigation (equal); methodology (equal); project administration (equal); resources (equal); software (equal); supervision (equal); validation (lead); visualization (lead); writing – original draft (lead); writing – review and editing (equal). **Stephanie Witczak:** Data curation (equal); formal analysis (supporting); funding acquisition (supporting); investigation (equal); methodology (equal); project administration (equal); resources (equal); writing – original draft (supporting); writing – review and editing (equal). **Adrian Aebischer:** Conceptualization (supporting); data curation (supporting); investigation (supporting); methodology (supporting); project administration (supporting); writing – original draft (supporting); writing – review and editing (supporting). **Valentijn van Bergen:** Data curation (equal); investigation (equal); project administration (equal); resources (equal); validation (equal); writing – review and editing (equal). **Benedetta Catitti:** Conceptualization (supporting); data curation (equal); investigation (supporting); project administration (equal); resources (equal); validation (equal); writing – review and editing (equal). **Martin U. Grüebler:** Conceptualization (equal); data curation (supporting); formal analysis (supporting); funding acquisition (lead); investigation (equal); methodology (equal); project administration (equal); resources (equal); supervision (lead); validation (supporting); visualization (supporting); writing – original draft (supporting); writing – review and editing (equal).

## FUNDING INFORMATION

This work was funded by the Swiss National Science Foundation (Grant 31003A_169668 to M. U. Grüebler).

## CONFLICT OF INTEREST

We declare that none of the authors is subject to any conflict of interest.

## Supporting information


Appendix S1

## Data Availability

All data used for the analyses is available on the vogelwarte.ch Open Repository: https://doi.org/10.5281/zenodo.7229211.
