# Supplementary material for: Determinants of departure to natal dispersal across an elevational gradient in a long‐lived raptor species
Source: Ecol Evol. 2023 Jan 16;13(1):e9603. doi: 10.1002/ece3.9603 (PMC9842906; doi:10.1002/ece3.9603)
Supplement: Supplementary file 1 — Appendix S1 [file ECE3-13-e9603-s001.docx]

# Electronic Supporting Information

# Determinants of departure to natal dispersal across an elevational gradient in a long-lived raptor species

Authors

Patrick Scherler^a,b,^*, Stephanie Witczak^a,b^, Adrian Aebischer^c^, Valentijn van Bergen^a^, Benedetta Catitti^a,b^ & Martin U. Grüebler^a^

Affiliations

^a^ Swiss Ornithological Institute, Sempach, Switzerland

^b^ Department of Evolutionary Biology and Environmental Studies, University of Zurich, Zurich, Switzerland

^c^ Fribourg, Switzerland, adaebischer@pwnet.ch

*corresponding author contact:

Dr. Patrick Scherler, Swiss Ornithological Institute, Seerose 1, CH-6204 Sempach

patrick.scherler@vogelwarte.ch

### S1: Validation of the temporal threshold for age at departure from natal home range

For juvenile red kites, we estimated individual departure from the natal home range based on a) a radius surrounding the nest, and b) a duration in which the individual had to stay outside the radius (analogous to method 7, Weston et al., 2013). A combination of a spatial and a temporal threshold assumes that once the individual stays outside the natal home range longer than the temporal threshold, it can be considered as definitely departed from the parental home range. We selected a spatial threshold of 2 km and a temporal threshold of 2 days. Nevertheless, we validated different temporal and spatial thresholds (Fig. S1.1). Correlations between departure age values calculated based on different spatial and temporal thresholds were estimated by using pairwise Spearman correlation tests (Fig. S1.1). We assessed the differences in age at departure from natal home range between different time thresholds by calculating the mean deviation ($\sqrt{\sum{(Residuals)}^{2}/n}$) and the mean bias (${(Residuals)}^{2}/n$) for every threshold value in relation to the benchmark method (2 days) (Table S1.1).

The different temporal thresholds showed similar patterns (Fig. S1.2) and the correlations between the departure age values for a time threshold of 2 days and of 1, 3 and 5 days were high (Spearman’s ρ > 0.85). Moreover, the bias of longer temporal thresholds was positive and the one of shorter temporal thresholds negative, as expected. Due to the high correlation between different temporal thresholds, we decided to use the benchmark method (2 km / 2 days) for further modelling.

Table S1.1: Estimated age at departure from natal home range based on varying temporal thresholds (time spent outside natal home range) and bias, deviation and pairwise correlation between different thresholds and the chosen method with a 2-day threshold.

| Temporal threshold | Departure age; median (range) | | Bias | Mean deviation | Spearman's ρ |
| --- | --- | --- | --- | --- | --- |
| 1 day | | 82 (48-148) | -3 | 39 | 0.93 |
| **2 days** | | **83 (61-187)** |  |  |  |
| 3 days | | 84 (61-187) | 2 | 35 | 0.92 |
| 5 days | | 86 (61-187) | 5 | 78 | 0.86 |
| 9 days | | 89 (61-208) | 12 | 182 | 0.74 |


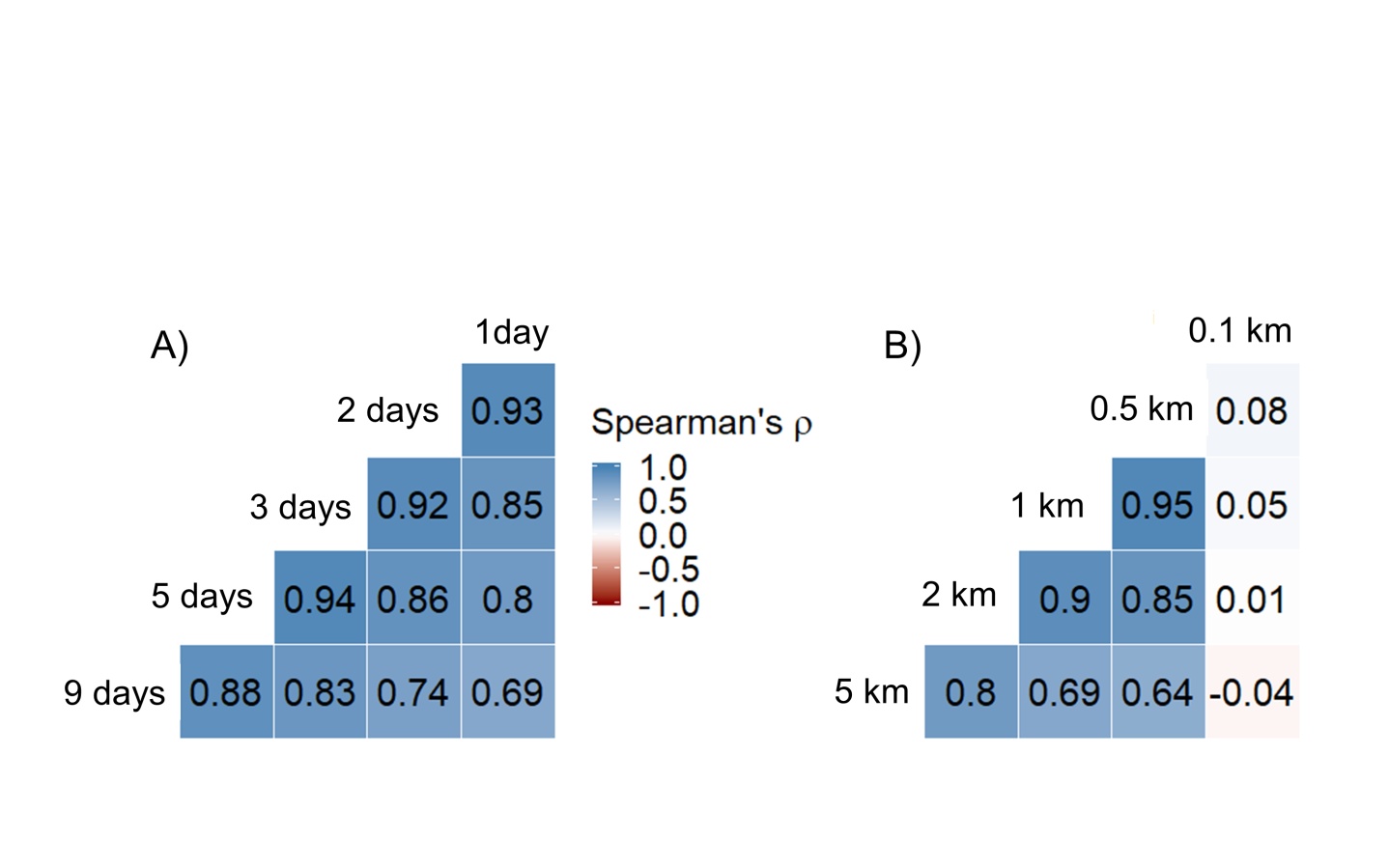


Fig. S1.1: Correlation (pairwise Spearman correlation) between A) different temporal thresholds for defining departure with a constant spatial threshold of 2 km and B) different spatial thresholds for defining departure with a constant temporal threshold of 2 days (48h).


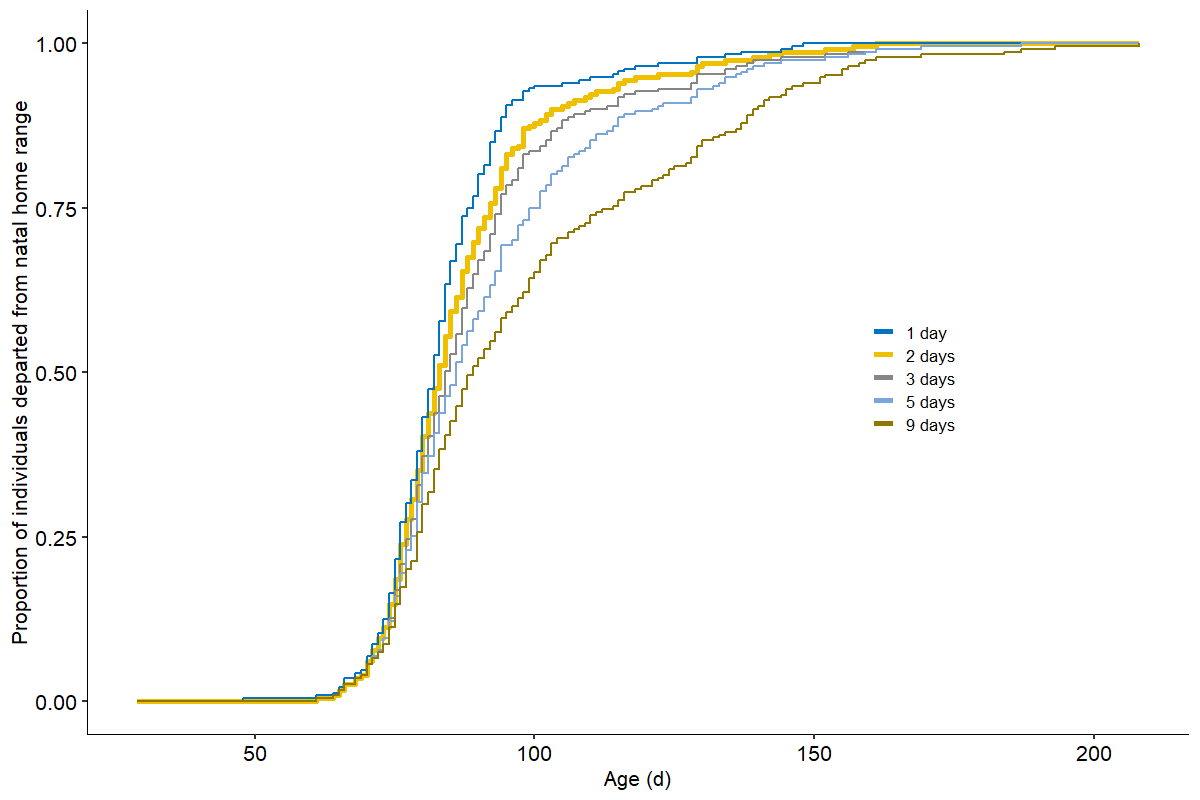


Fig. S1.2: Proportions of individuals departed from their natal home range in relation to age for different temporal thresholds (being outside a radius of 2 km around the natal site). A temporal threshold of two days (yellow line) was chosen for the analyses investigating factors affecting departure age.

### S2: Qualitative validation of departure age from full family movement trajectories

In order to qualitatively assess the exact timing of independence from parental care and to get an idea of (a) potential parental care outside the parental breeding home range, and (b) the pattern of change in juvenile behaviour that leads to the simultaneous increase in distance to the parents and the parental home range, movement trajectories of a subset of five families from three parental couples were analysed. For every juvenile, the distance to the closest parent and the nest was calculated in hourly intervals from tagging to mid-September (average migration date) in order to define the timepoint when the distance from the closest parental individual exceeded 2 km for more than 48 consecutive hours. We assumed that at this point the juvenile was able to sustain itself without contact to the parents. We visually inspected distances to the nest and to the closest parent for 10 juvenile red kites of five broods (see example Fig. S2). Before permanently staying more than 2 km from the closest parent for more than 48 hours, we observed 1-3 short excursions exceeding the distance of 2 km to the closest parent. These excursions preceding independence were short (few hours - one day) and individuals always returned to their natal home range. However, after these short explorative flights, the distance to the nest and the distance to the closest parent varied more often simultaneously which indicates that leaving the natal home range was not performed in company of parents and hence can be considered as a ‘true’ departure from the natal home range. The increase in distance to the nest and to parents was rapid and usually maintained at a high level for more than 48 hours. Visual inspection of the tracking data can exclude the possibility that adults left their home range together with their dependent offspring. These qualitative results provide further support for the use of the selected spatio-temporal threshold to identify departure of juveniles from natal home range, i.e. the onset of dispersal.


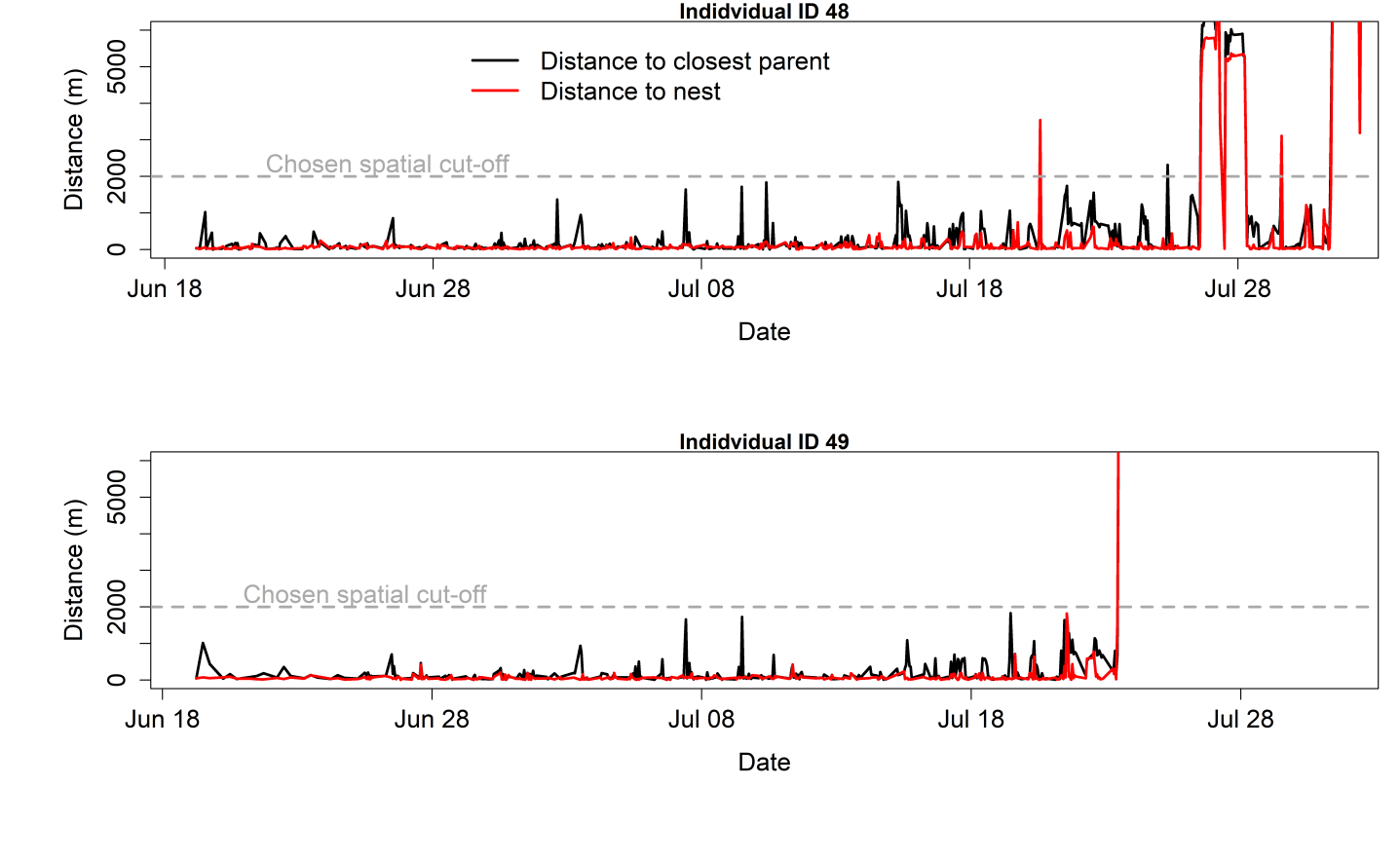
Fig. S2: Examples for the parallel pattern of distances from the parents (black) and from the nest (red) of two sibling individuals. When the spatial threshold of 2 km was exceeded for 48 subsequent hours, the bird was assumed to have departed from the parental home range to dispersal. Short explorative excursions prior to departure do not exceed this spatio-temporal threshold.

### S3: Evaluation of body condition effects on age at departure

Dispersal decisions are influenced by the context experienced, as well as by the morphological and physiological condition of the individual (Clobert, 2012). In our study we focussed on the role of natal habitat factors for departure decisions, and we disentangled the effect of food availability from the effects of other factors using a food supplementation experiment. However, food supplementation also affects the body mass of last-hatched fledgling individuals (Nägeli et al., 2022), and therefore body condition measured as growth corrected body mass might affect departure decisions. We decided to use only experimental treatment as explanatory variable in our main analyses investigating condition-dependent departure decisions, because (1) experimental food supplementation also drives physiological components of body condition not considered by body mass-based condition measures (see Catitti et al., 2022), and (2) complex interactions between nestling rank, nestling survival and food provisioning might mask the effect of feeding treatment on body condition, and thus on departure decisions. Here, we present important background information about these interactions and the potential role of body mass-based condition measures for departure decisions in our study system.

Methods: We calculated an index for fledgling body condition using mass-size residuals, by regressing body weight (g) on tarsus length (mm) for all individuals that survived to the pre-fledging climb (n = 311). In order to minimize potential growth bias associated with mass-size residuals (Green, 2001; Peig & Green, 2010), we visually examine the relationship between body weight, tarsus length and age to ensure trends in both metrics have levelled off by c. 35 to 45 days old. Therefore, we regressed body mass with tarsus length per sex, using a standardized major axis regression (‘sma’ in package smatr: Warton et al. (2012)) to account for potential error in both the x and y-axis variables.

To investigate the possible carry-over effect of food supplementation via body condition at fledging on departure age, we first analysed the effects of the feeding treatment and elevation on body condition in a linear mixed model. We also controlled for year, sex brood size at fledging and hatching rank. We included all possible two-way interactions and removed non-significant interactions in a stepwise procedure. No interactions remained in the model.

In a second step we investigated possible effects of body condition on age at departure by including body condition and all important explanatory variables from the main analysis as fixed factors (see Table 1) but excluding feeding treatment assuming that body condition reliably reflects the result of the experimental treatment. In both models we included brood ID as a random effect with restricted maximum-likelihood (REML) estimation of the associated variance component.

Results and discussion: In contrast to Nägeli et al. (2022), we found no clear effect of food supplementation on body mass-based body condition (Table S3.1). We also found no clear effect of body condition on departure age (Table S3.2). One reason for the contrasting results of a missing relationship between food supplementation and body condition might be the considerably smaller sample size in the present study. This is important as interacting effects of the feeding treatment can mask a clear relationship between supplementary feeding and increased body condition. In our study species, food supplementation during the nestling period considerably affected nestling survival and nestling growth rate during the phase of exponential growth, rather than the final body condition of nestlings of all ranks (Nägeli et al., 2022). Since body condition was reduced in large broods and food supplementation increased brood size by increasing survival of low-ranked nestlings, we were not able to find the expected effect of experimental treatment on body condition (Fig. S3), even though it likely exists. Nonetheless, feeding treatment strongly affected the stress response of red kite fledglings (Catitti et al., 2022). These results suggest that (1) physiological components of body condition rather than relative body mass are responsible for the condition-dependent departure presented in this study, and (2) using experimental treatment as explanatory variable in our main analyses represents an adequate approach to investigate condition-dependent dispersal patterns.

Table S3.1: Model estimates of the linear mixed effect model investigating factors affecting body condition (size corrected body mass). Brood ID was included as random effect (n = 150 individuals from 100 broods). Significant effects are highlighted in bold.

| **Variable** | **Estimate** | **SE** | **95% CrI** |
| --- | --- | --- | --- |
| Intercept | **0.70** | **0.16** | **0.39 – 0.99** |
| Feeding treatment [fed] | 0.24 | 0.16 | -0.09 – 0.57 |
| Hatching date | -0.05 | 0.09 | -0.23 – 0.12 |
| Hatching rank [2^nd^-4^th^ hatched] | 0.16 | 0.12 | -0.05 – 0.39 |
| Hatching rank [singleton] | -0.30 | 0.29 | -0.88 – 0.28 |
| **Fledging brood size** | **-0.31** | **0.14** | **-0.63 – -0.02** |
| **Elevation** | **0.20** | **0.08** | **0.04 – 0.36** |
| Sex [Male] | -0.12 | 0.11 | -0.32 – 0.10 |
| **Year [2017]** | **-1.23** | **0.17** | **-1.57 – -0.90** |
| Random effect variance [95% credible interval]: Brood 0.56 [0.50, 0.64] | | | |

Table S3.2: Model estimates of the linear mixed effect model investigating factors affecting age at departure. Potentially covarying effects with body condition were not included (feeding treatment and rank). Brood ID was included as random effect (n = 150 individuals from 100 broods). Significant effects are highlighted in bold.

| **Variable** | **Estimate** | **SE** | **95% CrI** |
| --- | --- | --- | --- |
| **Intercept** | **79.49** | **1.83** | **75.77 – 83.04** |
| **Hatching date** | **-2.43** | **1.01** | **-4.42 – -0.35** |
| Elevation | 0.91 | 1.07 | -1.10 – 3.03 |
| **Sex [Male]** | **3.57** | **1.53** | **0.78 – 6.61** |
| Distance to roosting site | -2.15 | 1.10 | -4.29 – 0.05 |
| Year [2017] | 4.36 | 2.32 | -0.16 – 8.81 |
| Territory density | 0.26 | 0.93 | -1.54 – 2.13 |
| Body condition | 0.98 | 1.03 | -0.99 – 2.94 |
| Random effect variance [95% credible interval]: Brood 3.81 [3.23, 4.50] | | | |


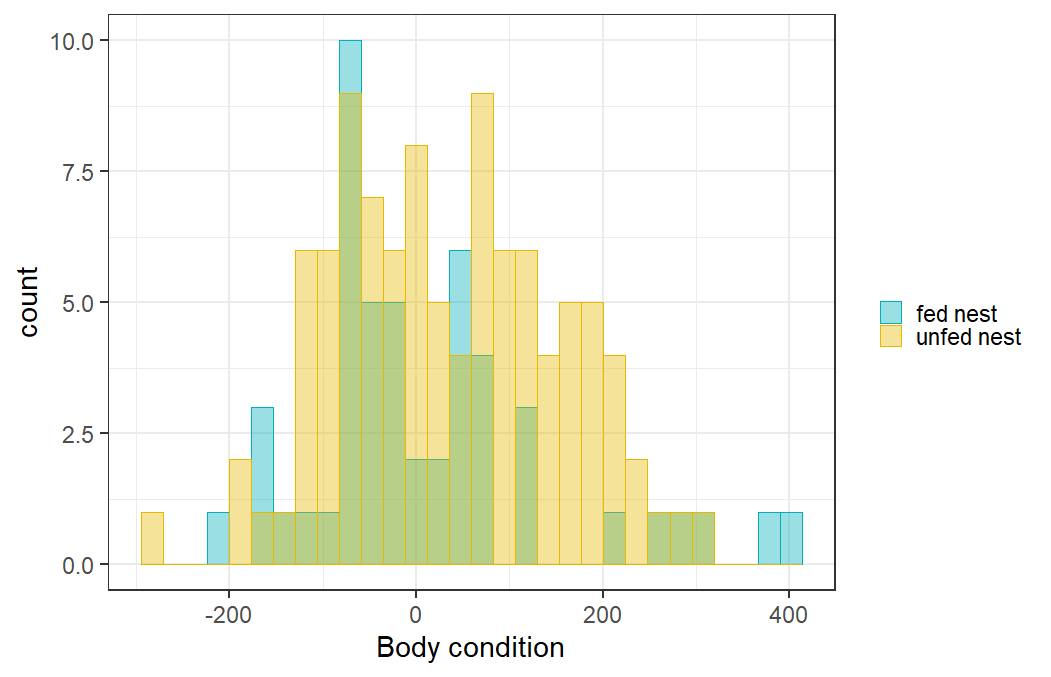


Fig. S3 Distribution of residual body condition values for supplementary fed (blue; n = 50, mean = 14.3 ± 134.6 SD) and control individuals (yellow; n = 108, mean = 34.4 ± 118.0 SD).

References

Catitti, B., Grüebler, M. U., Kormann, U. G., Scherler, P., Witczak, S., van Bergen, V., & Jenni-Eiermann, S. (2022). Hungry or angry? Experimental evidence for the effects of food availability on the stress response of developing wild raptor nestlings. *Journal of Experimental Biology*, *submitted*.

Clobert, J. (Ed.) (2012). *Dispersal ecology and evolution* (First edition). Oxford: Oxford Univ. Press.

Green, A. J. (2001). MASS/LENGTH RESIDUALS: MEASURES OF BODY CONDITION OR GENERATORS OF SPURIOUS RESULTS? *Ecology*, *82*(5), 1473–1483. https://doi.org/10.1890/0012-9658(2001)082[1473:MLRMOB]2.0.CO;2

Nägeli, M., Scherler, P., Witczak, S., Catitti, B., Aebischer, A., van Bergen, V., . . . Grüebler, M. U. (2022). Weather and food availability additively affect reproductive output in an expanding raptor population. *Oecologia*, *198*(1), 125–138. https://doi.org/10.1007/s00442-021-05076-6

Peig, J., & Green, A. J. (2010). The paradigm of body condition: A critical reappraisal of current methods based on mass and length. *Functional Ecology*, *24*(6), 1323–1332. https://doi.org/10.1111/j.1365-2435.2010.01751.x

Warton, D. I., Duursma, R. A., Falster, D. S., & Taskinen, S. (2012). smatr 3 - an R package for estimation and inference about allometric lines. *Methods in Ecology and Evolution*, *3*(2), 257–259. https://doi.org/10.1111/j.2041-210X.2011.00153.x

Weston, E. D., Whitfield, D. P., Travis, J. M. J., & Lambin, X. (2013). When do young birds disperse? Tests from studies of golden eagles in Scotland. *BMC Ecology*, *13*, 42. https://doi.org/10.1186/1472-6785-13-42
